# Duration of therapy and treatment compliance of depressive patients at clinical high-risk for psychosis

**DOI:** 10.1192/j.eurpsy.2022.1750

**Published:** 2022-09-01

**Authors:** M. Omelchenko

**Affiliations:** Mental Health Research Centre, Youth Psychiatry, Moscow, Russian Federation

**Keywords:** Clinical high-rick, lack of awareness, compliance, duration of treatment

## Abstract

**Introduction:**

It is known that early withdrawal can lead to a worsening of mental health. This is particularly relevant for depressive patients with clinical high-risk for psychosis (CHR) for whom the recommended duration of treatment has not been established.

**Objectives:**

Analyze the actual treatment duration of depressive patients at CHR after their discharge from hospital and compare it with the group of depressive patients without CHR.

**Methods:**

A comparative study of 124 depressive patients with CHR and 27 depressive patients without CHR was conducted within a year after discharge from hospital to assess the therapy duration and treatment compliance.

**Results:**

Within a year after discharge only 12.1% depressive patients with CHR and 29.6% ones without CHR continued to receive the therapy. The average duration of treatment after discharge was 7.4 months in the first group and 11.7 months in the second group. The majority of patients stopped treatment for the following reasons (in descending order of importance): lack of awareness of their mental condition (51.9% vs 40.3%), side effects (38.7% vs 11.1%), and negative attitudes towards the treatment on the part of patients’ immediate family members (8.9% vs 7.4%).

**Conclusions:**

It has been found out that depressive patients with CHR are less likely to follow medical prescriptions than ones without CHR, they are more likely to have the lack of awareness of their mental condition, they are more likely to have side effects of the therapy. These findings require the development of a universal approach to the treatment of such patients after discharge from hospital.

**Disclosure:**

No significant relationships.

